# A survey of healthcare professionals' knowledge of emergency oxygen use in adult patients

**DOI:** 10.1186/cc9568

**Published:** 2011-03-11

**Authors:** A Hartopp, K Horner, C Botfield

**Affiliations:** 1 Princess Royal University Hospital, Orpington, UK

## Introduction

There are many inaccurate teachings and a paucity of quality evidence about oxygen. We aimed to assess knowledge levels amongst healthcare professionals who administer oxygen with respect to basic physiology, delivery devices and the potential to cause harm in commonly encountered emergency situations.

## Methods

The salient clinical points from the British Thoracic Society guidance on Emergency Oxygen use in Adults Patients [1], as determined independently by three doctors, were incorporated into a questionnaire. The survey was conducted at a large district general hospital amongst frontline staff. Clinicians of all grades and backgrounds including emergency, surgical, anaesthetic and medical staff were surveyed under direct supervision.

## Results

A total of 196 people were surveyed, including 107 doctors (D), 69 nurses (N), 10 midwives (M) and 10 physiotherapists (P). Only 70% knew how to set up a non-rebreathe mask (D 62%, N 87%, P 80%, M 40%). Further, just 74% selected this as their first-line delivery device in a critically ill patient. For a simple facemask a flow rate of 5 to 101/minute is recommended (D 51%, N 54%, P 60%, M 90%), whilst the maximum flow rate by nasal cannulae is 61/minute, known by 14% of participants. Interestingly mouth breathing does not reduce the inspired oxygen concentration delivered by nasal cannulae, which was known by 37%. Recent evidence suggests the physiology of hypercapnic respiratory failure due to excessive oxygen therapy in some COPD patients is mainly due to worsening V/Q mismatching rather than a loss of hypoxic drive (D 16%, N 6%, P 0%, M 20%). In the absence of hypoxia, oxygen is not recommended in myocardial infarction (MI) or stroke because of hyperoxaemia-induced vasoconstriction. There was better awareness of oxygen use in stroke, with 41% answering correctly compared with 18% in MI. Of the vital signs, respiratory rate is the best predictor of severe illness (D 64%, N 71%, P 80%, M 70%). A >3% drop in saturations, even if within the normal range, is significant (D 83%, N 78%, P 60%, M 60%). Therefore oxygen should be titrated to a target saturation (D 47%, N 52%, P 40%, M 80%) rather than administering maximal oxygen therapy, which may mask acute deterioration.

## Conclusions

In our hospital there is a widespread lack of awareness about emergency oxygen. Patients are potentially being administered or deprived of oxygen in a manner detrimental to their care. Education is needed to protect patients and ensure correct teaching to future generations of medical professionals.
